# Magntic susceptibility as a proxy to heavy metal content in the sediments of Anzali wetland, Iran

**DOI:** 10.1186/1735-2746-9-34

**Published:** 2012-12-27

**Authors:** Mohammad Reza Vesali Naseh, Abdolreza Karbassi, Fereydoon Ghazaban, Akbar Baghvand, Mohammad Javad Mohammadizadeh

**Affiliations:** 1 Graduate Faculty of Environment, University of Tehran, Tehran, Iran; 2 Iran Department of Environment, Tehran, Iran

**Keywords:** Heavy metals, Magnetic susceptibility, Sediment cores, Cluster analysis, Anzali wetland

## Abstract

Heavy metal concentrations and magnetic susceptibility of sediment samples were analyzed as indicators of urban and industrial contamination in Anzali wetland in Gilan, Iran. The aim was to investigate the suitability of magnetic properties measurements for indicating heavy metal pollution. The concentration of six heavy metals (Ni, Cr, Cd, Zn, Fe, and Pb) was determined in different depths of four sediment core samples within four different regions of the wetland (Abkenar, Hendekhaleh, Shijan and Siakeshim). Average concentration of heavy metals in the sediment cores was higher than the severe effect level (SEL) for Ni, Cr and Fe (77.26, 113.63 ppm and 5.2%, respectively) and lower than SEL for Cd, Zn and Pb (0.84, 137.7, 29.77 ppm, respectively). It was found that the trend of metal concentrations with the depth is different in each core and is related to the pollution discharges into the rivers entering the wetland. Core magnetic susceptibility measurements also showed different magnetic properties in each core. Cluster analysis was applied using Pearson correlation coefficient between heavy metal concentrations and magnetic properties across each core. Significant relationship was found to exist between magnetic susceptibility and the concentration of Ni in Abkenar and the concentration of Fe in other regions. Whereas Abkenar is almost the isolated and uncontaminated region of the wetland, it revealed a difference in magnetic properties between contaminated and uncontaminated sediments. It was concluded that magnetic properties of samples from contaminated zone were mostly related to Fe content. The result of this study demonstrated that magnetic susceptibility measurements could be applied as a proxy method for heavy metal pollution determination in marine environments in Iran especially as a rapid and cost-effective introductory site assessments.

## Introduction

Magnetic susceptibility (MS) measurements are being used as an approximate tool for detecting industrial pollutions, because they are comparatively simple, fast and cost effective analyses. [1-7]). This method also could be applied as a tool for the assessment of heavy metal contamination in sediments [8], on the other hand as a proxy for heavy metal pollution. Petrovsky^′^ and Ellwood [6] discovered that magnetic susceptibility and Zn concentrations show very similar spatial distributions in a 20,000 m^2^ area at the Litavka River, Czech Republic, where ashes from a lead smelter are weathering in the fluvisols. Chan *et al*., [2], revealed that a significant correlation exists between the magnetic susceptibility and the concentration of Pb, Zn and Cu as well as Tomlinson pollution load index (PLI) in seabed sediments of Hong Kong Harbour. Schmidt et al., [9], investigated the suitability of field magnetic measurements for indicating heavy metal pollution. Geochemical analysis of their soil samples from Bradford, England, showed close correlation of concentrations between Fe, Cu, Mn, and Ni. In addition, Fe concentrations correlated with magnetic susceptibility field measurements. The results of their study demonstrated the potential of magnetic susceptibility field mapping for fast preliminary site assessment, greatly reducing the scale of subsequent geochemical sampling and analysis.

Magnetic susceptibility measurements, in Iran, have been applied for survey on Caspian sea-level fluctuation [10], but as a proxy for industrial contamination has been employed only in urban topsoils in the arid region of Isfahan [5]. They measured the magnetic susceptibility of 113 collected soil samples from public parks and green strips along the rim of roads with high-density traffic within the city of Isfahan using the Bartington MS2 dual frequency sensor. As, Cd, Cr, Ba, Cu, Mn, Pb, Zn, Sr and V concentrations were also measured in all collected soil samples. They discovered that Pb , Cu, Zn, and Ba have positive significant correlations with magnetic susceptibility, but As, Sr, Cd, Mn, Cr and V have no significant correlation with the magnetic susceptibility. There was also a significant correlation between pollution load index (PLI) and magnetic susceptibility. Finally they indicated the potential of the magnetometric methods to evaluate the heavy metal pollution of soils.

The present study is trying to investigate the suitability of magnetic properties measurements for indicating heavy metal pollution in Anzali wetland at the north of Iran. The result of this study suggests a useful, fast and cost-effective method for assessment of environmental pollutions in Iran.

## Materials and methods

### Study area

**Figure 1:**
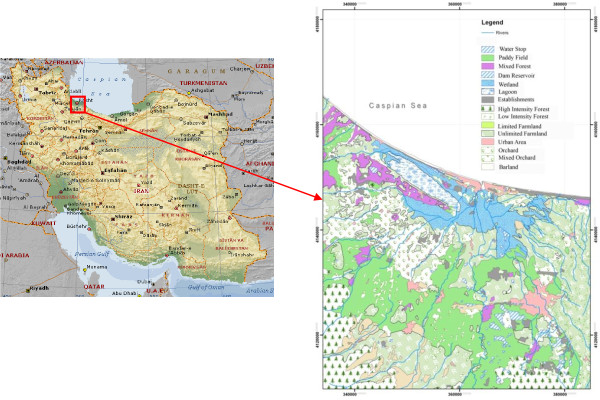
Anzali wetland location and landuse of its basin.

**Figure 2:**
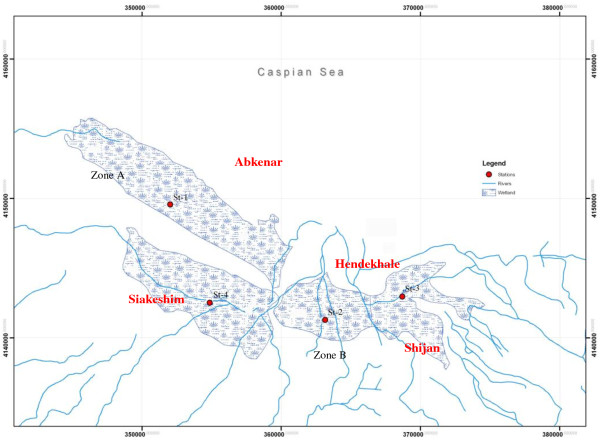
Anzali wetland regions and sampling sites.

Anzali wetland is a large complex environment of fresh water lagoons with extensive reed-beds, shallow impoundments and seasonal flooded meadows. It is extremely important as a spawning and nursery ground for fish, and as a breeding, staging and wintering area for a wide variety of waterfowl. It is located in the northern part of Iran, along the coast of the Caspian Sea approximately at north latitude between 37o 25^′^ and 37o 32^′^ and east longitude between 49o 15^′^ and 49 o 36^′^. It has a catchment area of 3610 km2. Approximately 42% of the catchment area is covered by forests. Among the landuse categories, forest has the largest share of 42%, followed by paddy field/farmland (26.7%) and orchard (8.6%) in that order (Figure 1). There are 10 major river systems entering the wetland and some of them have large discharges of urban and industrial wastewater along their way. The annual mean discharge into the wetland is estimated at 76.14 m^3^/s and the average COD is about 26.5 mg/L. The average annual precipitation of Anzali wetland watershed is about 1200 mm. Considering inflowing rivers, the wetland can be divided to 2 zones (Figure 2): west region with only one river inflow (Zone A), and the other regions with 9 rivers inflows (Zone B).

### Sampling locations

The Anzali wetland is shaped from 4 regions: west region (Abkenar), central region (Hendekhaleh), Siakeshim and east region (Shijan). Sampling sites were chosen approximately in the center of each region to represent the situation of each part (Figure 2): St1 in Abkenar, St2 in Hendekhaleh, St3 in Shijan and St4 in Siakeshim. Situation of sampling point in some locations (low depth marsh areas) was also considered for ease of sampling.

### Sampling methods

Sediment cores were collected in slosh mode using a piston gravity corer in May 2011. The core lengths were 70, 70, 80 and 50 cm and the diameter was 6 cm. All the samples were sealed by nylon and transferred to sediment laboratory of Iranian National Institute for Oceanography, Tehran, Iran, for magnetic susceptibility analysis. For geochemical analyses, they were transferred to sediment and chemistry laboratory of Water Research Institute, Tehran, Iran. After the polyethylene tube was cut off carefully, the sediment columns were sectioned into slices in depths of 0, 2, 6, 10, 15, 30, 50 and 70 cm along core 1 and core 2; 0, 2, 6, 10, 15, 20, 40, 60 and 80 cm along core 3 and 0, 2, 6, 10, 15, 30 and 50 cm along core 4.

### Magnetic susceptibility measurements

Magnetic susceptibility (MS) is a measure that particular sediments are magnetized when subjected to a magnetic field. The ease of magnetization is ultimately related to the concentration and composition (size, shape and mineralogy) of magnetizable material contained within the sample. Any sediment core possessing downcore variation per unit volume in the concentration and composition of magnetizable minerals will yield a MS curve reflecting these changes [11].

Magnetic susceptibility measurements are a non-destructive and cost effective method of determining the presence of iron-bearing minerals within the sediments. The whole cores, or individual sediment samples, are exposed to an external magnetic field which causes the sediments to become magnetized according to the amount of Fe-bearing minerals present in the samples.

In our system, using Bartington MS2 System [11] whole cores are moved incrementally (generally in 1 cm) by a track motor through a susceptibility loop (of varying size) in which a magnetic field is generated and which magnetizes the sample susceptible substances (minerals or mineraloids) within the sediment. Samples that are rich, per unit volume, in magnetizable substances will yield high readings.Samples that are poor in magnetizable substances, or contain diamagnetic minerals, will yield lower or negative values.

### Geochemical analysis

Subsamples for geochemical analysis were chosen incrementally in different depths along core samples, dried and powdered in agate mortar. Digestion of organic matter and dissolution of silicates for total elemental analysis were done as described below: 1.0 g of the 100-mesh (0.15 mm) sediment was weighed into a 100-mL Teflon beaker and 10 mL of HNO_3_ and 10 mL of HClO_4_ were added. The beaker was covered with a Teflon watch cover and heated at 200°C for 1 h. The cover was removed and heating was continued until the volume became 2 to 3 mL. After cooling the sample, 5 mL of HClO_4_ and 10 ml of HF were added; Teflon cover was put and heated at 200°C until all siliceous materials had been dissolved. Then the cover removed and heating continued until the volume was 2 to 3 mL. The digest was cooled, 10 mL of 50% HCl was added, Teflon cover put and heated at 100°C for 30 min. After cooling the sample brought to 50-mL volume. The solution is then ready for ICP determination [12]. The concentrations of heavy metals were determined by Varian 710-ES Inductively Coupled Plasma Mass Spectrometry (ICP-MS) according to APHA AWWA, WEF [13]. Each sample was duplicated and the average was reported.

### Pollution assessment

To assess metal concentrations in sediment, the New York State Department of Environmental Conservation [14] guideline was applied. It proposed the lowest effect screening levels (LEL) for Ni, Cr, Cd, Zn, Pb, and Fe of 31, 26, 0.6, 120, 31 mg/kg and 2%, respectively, and severe effect screening levels (SEL) of 75, 110, 9, 270, 110 mg/kg, and 4%, respectively. The pollution extent was assessed by two threshold values of LEL and SEL. If the LEL was exceeded, the metal could moderately impact biota health. If the SEL was exceeded, the metal could severely impact biota health [15].

## Results

### Geochemical results

**Figure 3:**
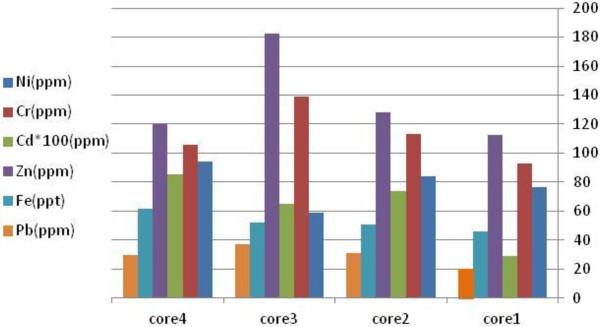
Comparison between average values of heavy metal concentrations in sediment cores.

Table 1 shows the concentration of heavy metals in subsamples in different depths and the average value of each core. Total average values of Ni, Cr, Cd, Zn, Pb, and Fe were 76.91, 113.74, 0.84, 137.98, 29.74 ppm and 5.2%, respectively. Comparison of the average value of heavy metal concentrations in sediment cores is illustrated in Figure 3. It can be observed that the values related to core 1 is lower than the other cores. Whereas core 1 represents Abkenar region, it could be resulted that this part of the wetland is less contaminated than the other parts. This fact could be deduced from rivers entering the wetland. As described in section 2.1 (Figure 2) only one river enters the west part of the wetland and the other rivers which are carrying urban and industrial wastewaters enter the other parts of the wetland.

**Table 1:** Concentration of heavy metals in subsamples

**Core**	**depth(cm)**	**heavy metals**					
		**Ni(ppm)**	**Cr(ppm)**	**Cd(ppm)**	**Zn(ppm)**	**Fe(%)**	**Pb(ppm)**
**Core 1 (Abkenar)**	0	67.30	73.70	0.24	95.90	4.86	16.30
	2	70.20	76.20	0.26	102.30	4.73	18.80
	6	75.60	85.20	0.32	120.60	4.85	22.60
	10	77.20	98.60	0.38	125.00	4.64	23.80
	15	79.10	108.50	0.32	129.60	4.55	24.80
	30	80.30	103.50	0.28	124.20	4.76	21.90
	50	82.50	98.60	0.26	102.30	4.35	18.80
	70	80.30	96.30	0.24	98.30	4.28	16.20
	average	76.56	92.58	0.29	112.28	4.63	20.40
**Core 2 (Hendekhaleh)**	0	64.20	101.55	0.74	135.30	5.82	16.30
	2	75.60	109.20	0.65	130.20	5.26	23.50
	6	68.30	116.30	0.85	132.80	5.53	25.60
	10	79.20	110.20	0.96	130.40	6.28	29.30
	15	88.50	136.80	0.88	125.60	5.36	32.80
	30	94.00	121.00	0.95	126.80	4.62	44.40
	50	105.00	108.60	1.35	118.50	3.03	25.10
	70	98.00	102.60	1.12	124.70	4.55	50.10
	average	84.10	113.28	0.94	128.04	5.06	30.89
**Core 3 (Shijan)**	0	60.00	98.80	3.64	101.70	5.67	35.77
	2	44.00	163.00	0.85	273.98	5.22	37.11
	6	85.50	159.50	2.43	224.82	5.78	27.23
	10	63.50	147.50	2.18	224.70	5.13	23.16
	15	43.00	128.00	1.74	243.50	4.96	61.78
	20	55.50	129.00	0.81	164.40	3.74	47.44
	40	63.00	113.00	0.70	156.97	4.71	18.73
	60	45.50	149.00	0.49	125.27	5.48	48.18
	80	70.00	162.00	0.38	128.03	5.97	53.50
	average	58.89	138.87	1.47	182.60	5.18	39.21
**Core 4 (Siakeshim)**	0	80.90	98.00	0.62	120.80	6.09	13.90
	2	84.20	101.20	0.56	119.30	6.23	22.30
	6	90.30	103.50	0.49	118.50	6.63	26.50
	10	91.50	96.30	0.52	115.30	6.95	28.30
	15	96.20	105.80	0.58	112.30	6.36	34.20
	30	102.40	108.50	0.52	110.80	5.83	36.50
	50	111.50	126.20	0.46	143.50	5.01	46.96
	average	93.86	105.64	0.54	120.07	6.16	29.81
Min		43.00	73.70	0.24	95.90	3.03	13.90
Max		111.50	163.00	3.64	273.98	6.95	61.78
Average		76.91	113.74	0.84	137.98	5.20	30.35
SEL		50.00	110.00	9.00	270.00	4.00	110.00

### Nickle contamination

The average value of Ni concentration was above SEL (50 mg/kg) at all cores. The maximum Ni concentration appeared at the depth of 50 cm at core 4 (Siakeshim), which was more than two times the SEL. A relatively constant Ni concentration was detected across core 1 (Abkenar), but in core 2 it increases with the depth increase to concentration of 105 ppm at the depth of 50 cm and decreases to 98 ppm at the depth of 70 cm. The minimum value of Ni concentration appeared at the depth of 15 cm in core 3.

### Choromium contamination

At core 2 and core 3 (Hendekhale and Shijan), the average value of Cr concentration was above SEL (110 mg/kg) and the maximum value appeared at the depth of 2 cm at core 2. In core 1 and core 4, the average Cr concentrations were below SEL and above LEL (26 mg/kg). The minimum value of Cr concentration appeared at the surface of core 1.

### Cadmium contamination

All of the Cd concentrations were below SEL (9 mg/kg) but the average values of Cd concentration in core 2 and core 3 were above LEL (0.6 mg/kg). The maximum concentration appeared at the surface of core 3 and the minimum value appeared at core 1.

### Zinc contamination

Zn concentration in all subsamples was below SEL (270 mg/kg) except for core 2 at the depth of 2 cm. the average value of Zn concentration was near LEL (120 mg/kg) in core 1, core 2 and core 4 and the minimum value appeared at the surface of core 1.

### Lead contamination

The average concentration of Pb in all sediment columns was below LEL (31 mg/kg) except for core 3 (Shijan), which was above LEL and below SEL (110 mg/kg). A relatively constant Pb concentration was detected across core 1 (Abkenar) but in core 4 it increases with the depth increase. The maximum value of Pb concentration appeared at the depth of 15 cm in core 3 and the minimum value appeared at the surface of core 4.

### Iron contamination

All of the average values for Fe percentage in sediment columns were above SEL (4%) and the maximum value of appeared at the depth of 10 cm in core 4. A relatively constant Fe concentration was detected across core 1, core 3 and core 4.

**Figure 4:**
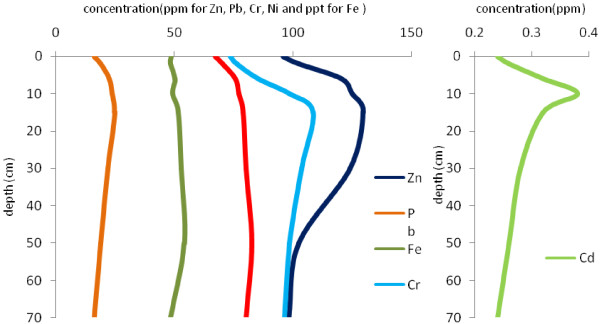
Variation of heavy metal concentrations with depth in core 1.

**Figure 5:**
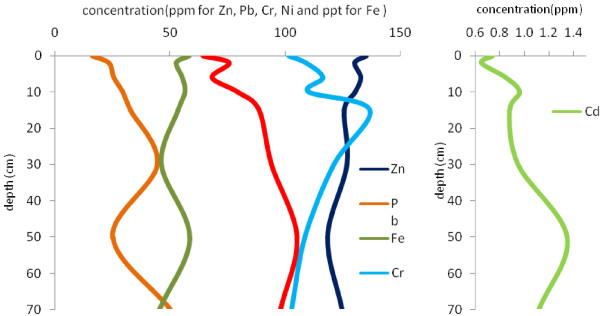
Variation of heavy metal concentrations with depth in core 2.

**Figure 6:**
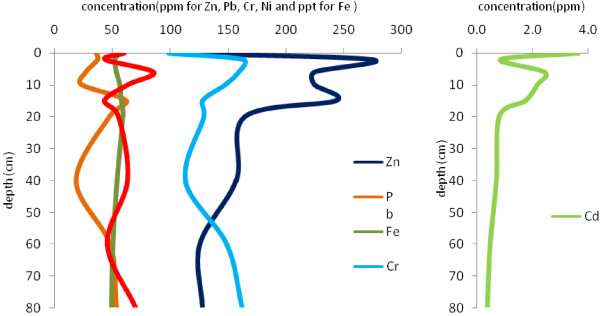
Variation of heavy metal concentrations with depth in core 3.

**Figure 7:**
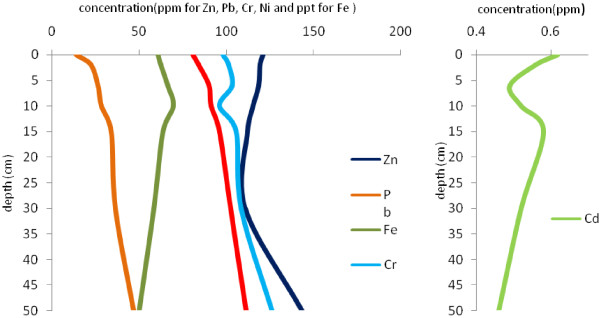
Variation of heavy metal concentrations with depth in core 4.

Figures 4,5,6,7 show the variation of heavy metal concentrations across core 1 to core 4 respectively. It can be observed that there is no distinct trend for concentration with the depth in sediment columns especially for core 2 and core 3. In core 1 as illustrated in Figure 4, the concentration of Pb, Cr, Zn and Cd is increasing first, and then decreases through the core depth. Fe and Ni concentrations have a relatively constant variation with the depth. In core 4, the concentration of Pb, Ni and Cr have increasing variation with the depth. Zn concentration decreases to the depth of 30 cm and increases to the end of the column. These variations are related to contaminations which have entered to the wetland during recent years.

### Magnetic susceptibility results

**Figure 8:**
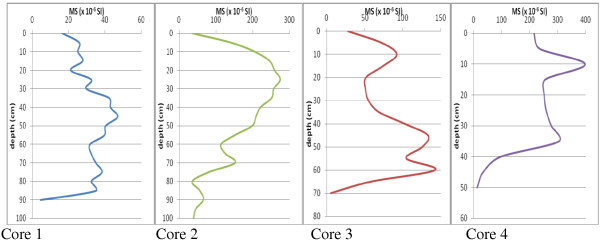
Magnetic susceptibility curve for core 1 to core4.

Magnetic susceptibility curves (MS curves) of core 1 to core 4 are illustrated in Figure 8. Variation of magnetic susceptibility with depth in core 1 is increasing to the middle depth and decreases to the end of the core. In core 2 magnetic susceptibility increases to depth of 25 cm and decreases to the end. Core 3 has three peaks in depths of 10, 45 and 60 cm and finally core 4 has two peaks in depth of 10 cm and 35 cm.

## Discussion

Table 2 compares the results of this study with previous studies on the Anzali wetland and some other lakes in the world. It should be explained that the values in this table is related to surface sediments and the values related to this study are the mean value of the depths of 0 and 2 cm of all sediment cores. Concentration of Ni was higher than the values of other lakes but comparable to the findings from previous studies on Anzali wetland. Cr, Cd and Fe concentrations in this study was near to the values of previous study on the wetland. Zn concentration was lower than the values of other lakes and comparable to Anzali wetland previous researches. Pb concentration was lower than previous studies on Anzali wetland and between the values of other lakes.

**Table 2:** Comparison of Ni, Cr, Cd, Zn, Fe and Pb concentrations in Anzali wetland and some other water bodies

**Site**	**Ni(ppm)**	**Cr(ppm)**	**Cd(ppm)**	**Zn(ppm)**	**Fe(%)**	**Pb(ppm)**	**Reference**
Anzali Wetland, Iran	68.3	102.7	0.94	134.9	5.49	23	Present study
Anzali Wetland, Iran	---	---	1.32	---	---	24.2	[16]
Anzali Wetland, Iran	75.7	100.1	0.31	125.4	5.2	26.1	[17]
Anzali Wetland, Iran	52.6	---	1.2	287.5	---	51.8	[18]
Avsar Dam Lake, Turkey	29.1	13.9	0.76	---	2.4	3.24	[19]
Storm water retention pond, New York, USA	13	8	---	300	---	110	[15]
Kolleru Lake, India	2	78	---	714	---	6	[20]
J. A. Alzate Reservoir, Mexican	43	145	---	233	---	76	[21]

The results of heavy metal concentrations across sediment columns were compared with the results of research carried out by Ghazban and Zare on the Anzali wetland in Table 3[17]. It can be observed that the results were relatively similar to the present study and there was not any distinct trend for the variation of heavy metal concentrations with the depth in sediment columns. This fact is related to the industrialization and urbanization of the wetland basin. There are 23 large plants in the wetland basin which only four of them have appropriate working wastewater treatment plant. Six of the other plants do not have proper wastewater treatment system and the other plants do not have any wastewater treatment plant [22]. Uncontrolled wastewater discharges from these plants to the rivers entering the wetland, resulted in higher heavy metals depositing in the wetland sediments during recent years. Whereas these pollutions do not emit to the rivers continuously, no clear trend could be detected for heavy metal contents with the depth. Except for industrial emission, some of these metals have considerable traffic related sources like Pb [23]. With the rapid process of urbanization, the number of automobiles has increased and gasoline discharges to the receiving water bodies led to heavy metals depositing in the wetland sediments. By relating the industrialization and urbanization process to the vertical distribution curves at these sediment cores, it is believed that the high trace metal concentrations of sediment in Anzali wetland result from rapid urbanization and industrialization, and lack of wastewater treatment.

**Table 3:** **Concentration of heavy metals in subsamples **[17]

**Region**	**depth(cm)**	**heavy metals**				
		**Ni(ppm)**	**Cr(ppm)**	**Zn(ppm)**	**Fe(ppt)**	**Pb(ppm)**
**Abkenar**	0	77.50	96.00	109.00	4.90	21.50
	5	78.00	90.00	107.00	5.15	21.80
	10	75.50	96.00	109.00	5.10	20.90
	20	79.30	97.00	114.00	5.20	19.20
	50	79.90	95.00	104.00	5.00	19.30
	average	78.04	94.80	108.60	5.07	20.54
**Hendekhaleh**	0	71.10	97.00	131.00	5.00	30.20
	5	67.80	102.00	125.00	4.65	29.60
	10	72.10	106.00	132.00	4.80	29.30
	20	69.90	104.00	117.00	4.90	29.80
	50	72.40	92.00	118.00	5.20	24.70
	average	70.66	100.20	124.60	4.91	28.72
**Shijan**	0	78.40	99.00	122.00	5.20	30.40
	5	81.00	101.00	129.00	5.80	23.40
	10	83.20	103.00	132.00	5.90	23.40
	20	79.10	100.00	125.00	5.25	22.20
	50	70.90	98.00	111.00	5.15	22.20
	average	78.52	100.20	123.80	5.46	24.32
**Siakeshim**	0	69.50	108.00	130.00	5.70	26.40
	5	80.40	97.00	126.00	5.60	24.90
	10	79.90	99.00	127.00	5.45	24.20
	20	73.00	99.00	109.00	4.95	22.20
	50	63.70	90.00	100.00	4.85	22.10
	average	73.30	98.60	118.40	5.31	23.96
Min		63.70	90.00	100.00	4.65	19.20
Max		83.20	108.00	132.00	5.90	30.40
Average		75.13	98.45	118.85	5.19	24.39

**Figure 9:**
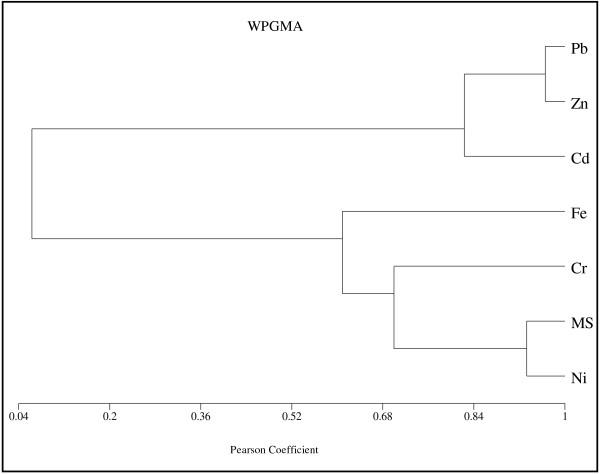
Cluster Analysis of MS and heavy metals for core 1.

**Figure 10:**
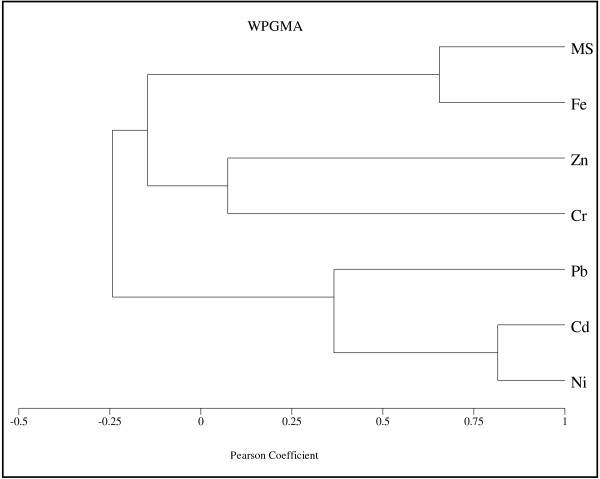
Cluster Analysis of MS and heavy metals for core 2.

**Figure 11:**
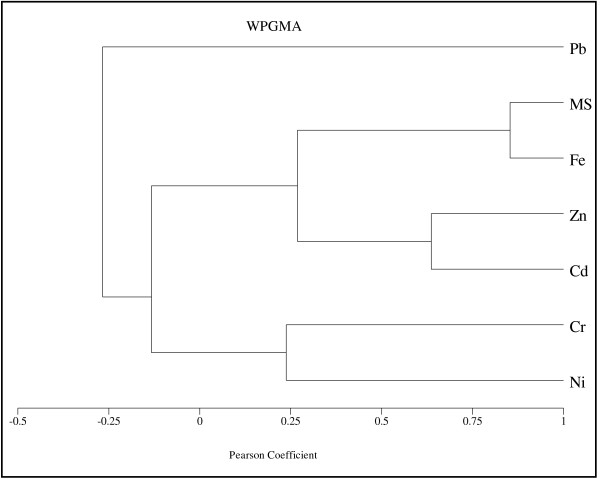
Cluster Analysis of MS and heavy metals for core 3.

**Figure 12:**
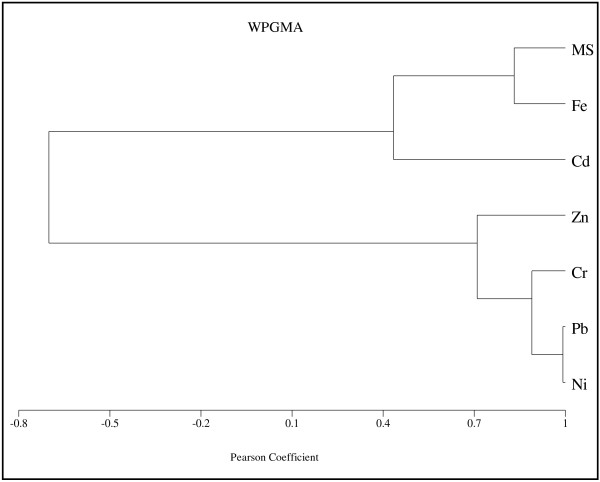
Cluster Analysis of MS and heavy metals for core 4.

To analyse the relationship between magnetic susceptibility and the concentration of each heavy metal along the cores, the cluster analysis was applied using Pearson correlation coefficient. Figures 9,10,11,12 show the dendrogram of magnetic susceptibility and heavy metals for core 1 to core 4 respectively. In Figure 9 there is strong relationship between MS and Ni and a relatively strong relationship for MS with Cr and Fe in core 1. It means that magnetic properties of core 1 are related to Ni mostly. There is also a good correlation between Pb, Zn and Cd. Figure 10 indicates a relatively strong relationship between MS and Fe in core 2, so it can be concluded that magnetic properties of core 2 are related to Fe content. In this core there is close correlation between Cd and Ni. Figures 11 and 12 show strong relationship between MS and Fe in core 3 and core 4. therefore their magnetic properties are related to Fe content. There is also close correlation between Cr, Pb and Ni in core 4. Close correlation between heavy metals signifies that they have originated from similar contaminant sources [24].

## Conclusion

The main objective of this study was to investigate the relationship between the magnetic susceptibility and the contamination of heavy metals in sediments of Anzali wetland. To achieve the aim, four stations in the wetland were chosen considering four different regions of the wetland and core sediment samples were collected. Six heavy metals (Ni, Cr, Cd, Zn, Fe, and Pb) were analyzed across each sediment core by geochemical analysis. Whole-core magnetic susceptibility measurements were done on each sample using Bartington MS2C System. To discover the relationship between magnetic susceptibility and heavy metals content, cluster analysis was applied using Pearson correlation coefficient. Major findings are listed below:

High trace metal concentrations of sediment in Anzali wetland result from rapid urbanization and industrialization, and lack of wastewater treatment plants in surrounding industries. Whereas pollutions haven’t emitted to the wetland continuously, no clear trend could be detected for heavy metal contents in vertical distribution curves at these sediment cores.

Geochemical analysis of soil samples showed different correlations of concentrations in each core: in core 1 there was close correlation between Cd, Pb and Zn; in core 2 there was close correlation between Cd and Ni and in core 4 there was close correlation between Cr, Pb and Ni.

Significant relationship was found to be between magnetic susceptibility and the concentration of Fe in most of core samples. It concluded that magnetic properties of core samples were related to Fe content.

In west part of the wetland, Abkenar region (zone A in Figure 2), the relationship between MS and heavy metals was different with the other parts (zone B in Figure 2). It could be related to the contamination level of each zone. zone A is relatively isolated part of the wetland (only one river inflow) and consequently is less contaminated than zone B. comparison of average heavy metal contents in four sediment cores (Figure 3) confirmed this fact. It can be deduced from this finding that during last decades, before urbanization and industrialization of the wetland basin, the correlation of MS and heavy metals in Anzali wetland have been significant for Ni, Cr and Fe, but during recent years by rapid process of urbanization and industrialization and increasing contamination from rivers inflowing the wetland, this correlation had become significant for Fe.

The result of this study demonstrated that magnetic susceptibility measurements could be applied as a proxy method for heavy metal pollution determination in marine environments in Iran especially as a rapid and inexpensive preliminary site assessment. Such a survey should be accompanied by geochemical data for more accuracy. Although availability of suitable cores is very important in this application, if provided, magnetic susceptibility can offer scientists and engineers a quick and cost-effective tool of surveying seabed contamination by heavy metals.

## Competing interests

The authors declare that they have no competing interests.

## Authors’ contributions

The overall implementation of this study including design, experiments and data analysis, and manuscript preparation were the results of efforts by Corresponding author. All authors have made extensive contribution into the review and finalization of this manuscript. All authors read and approved the final manuscript.
